# Breaking Barriers in Thyroid Cytopathology: Harnessing Deep Learning for Accurate Diagnosis

**DOI:** 10.3390/bioengineering12030293

**Published:** 2025-03-14

**Authors:** Seo Young Oh, Yong Moon Lee, Dong Joo Kang, Hyeong Ju Kwon, Sabyasachi Chakraborty, Jae Hyun Park

**Affiliations:** 1 Terenz Co., Ltd., Busan 48060, Republic of Korea; osyoung540@gmail.com (S.Y.O.); dalant13@gmail.com (D.J.K.); 2 Department of Pathology, College of Medicine, Dankook University, Cheonan 31116, Republic of Korea; 12200301@dankook.ac.kr; 3 Department of Pathology, Wonju Severance Christian Hospital, Wonju College of Medicine, Yonsei University, Seoul 03722, Republic of Korea; kokalcon@yonsei.ac.kr; 4 Department of Surgery, Wonju Severance Christian Hospital, Wonju College of Medicine, Yonsei University, Wonju 26492, Republic of Korea

**Keywords:** papillary thyroid cancer, deep learning, convolutional neural network, multiple instance learning

## Abstract

Background: We address the application of artificial intelligence (AI) techniques in thyroid cytopathology, specifically for diagnosing papillary thyroid carcinoma (PTC), the most common type of thyroid cancer. Methods: Our research introduces deep learning frameworks that analyze cytological images from fine-needle aspiration cytology (FNAC), a key preoperative diagnostic method for PTC. The first framework is a patch-level classifier referred as “TCS-CNN”, based on a convolutional neural network (CNN) architecture, to predict thyroid cancer based on the Bethesda System (TBS) category. The second framework is an attention-based deep multiple instance learning (AD-MIL) model, which employs a feature extractor using TCS-CNN and an attention mechanism to aggregate features from smaller-patch-level regions into predictions for larger-patch-level regions, referred to as bag-level predictions in this context. Results: The proposed TCS-CNN framework achieves an accuracy of 97% and a recall of 96% for small-patch-level classification, accurately capturing local malignancy information. Additionally, the AD-MIL framework also achieves approximately 96% accuracy and recall, demonstrating that this framework can maintain comparable performance while expanding the diagnostic coverage to larger regions through patch aggregation. Conclusions: This study provides a feasibility analysis for thyroid cytopathology classification and visual interpretability for AI diagnosis, suggesting potential improvements in patient outcomes and reductions in healthcare costs.

## Simple Summary

Thyroid cancer is a common type of cancer that can be diagnosed by examining the cells of the thyroid gland. However, the current diagnostic system has some limitations, such as subjectivity and uncertainty. In this paper, we propose an artificial intelligence (AI)-driven framework to analyze the whole-slide images (WSIs) of 151 cases of thyroid cancer and provide highly accurate diagnoses for individual patch-level regions. We develop a backbone network that is strongly trained on regions of various sizes and that can achieve high performance in predicting the malignancy of thyroid cancer. Additionally, we show an uncertainty analysis that mimics the decision-making process of pathological experts, enabling the model to offer interpretable and confidence-weighted predictions. By integrating these elements, our approach offers a robust diagnostic technique for providing more information on thyroid malignancy to doctors and patients.

## 1. Introduction

In recent years, the incidence of papillary thyroid carcinoma (PTC), the most common type of thyroid cancer, has been on the rise globally. Fine-needle aspiration cytology (FNAC) has become an essential preoperative diagnostic modality for PTC. However, the current diagnostic systems for PTC have limitations, which can be addressed through the application of artificial intelligence (AI).

The Bethesda System for Reporting Thyroid Cytopathology (TBS) provides a standardized approach for interpreting FNAC results by categorizing them into six diagnostic categories based on the likelihood of malignancy. Nonetheless, the interpretation of TBS categories can be subjective, leading to interobserver variability, and there can be overlaps between categories. For instance, certain cytological findings may fall between categories such as “III. Atypia of Undetermined Significance (AUS)” and “V. Suspicious for Malignancy (SFM)”, which can lead to varying interpretations by different pathologists [1,2,3]. Such subjectivity and category overlap are inherent limitations of TBS, which may hinder accurate and reliable assessment. Furthermore, TBS categories do not provide localized diagnostic information from digital pathology slides, which limits their ability to offer detailed insights into specific areas of the thyroid cell. Generally, Bethesda categories are assigned to each whole-slide image (WSI) as a single diagnosis, which makes it difficult to perform patch-level diagnosis for specific regions. Labeling individual patches for each small region is impractical due to the labor-intensive and costly nature of the process. The lack of regional specificity makes it challenging to assess the risk of malignancy (ROM) in different regions, which is crucial for making accurate diagnoses. To overcome these limitations, our study proposes two deep learning diagnostic frameworks based on the convolutional neural network (CNN) and multiple instance learning. The first proposed framework focuses on patch-level classification, utilizing a task-specific (thyroid-cytopathology-specific) CNN architecture and is designed to accurately train on cytopathological features indicative of localized thyroid cancer, enabling the model to capture critical patterns related to malignancy. The second one leverages an attention-based deep multiple instance learning (AD-MIL) approach, combining a feature extractor based on the same CNN structure with an attention mechanism. This allows the model to effectively integrate information from small-patch-level regions and aggregate these features into higher-level predictions for larger, contextual regions, referred to as “bag-level” predictions. By expanding predictions from smaller to larger regions, we increase the diagnostic coverage, enabling more comprehensive and accurate identification of malignant areas within thyroid cytopathological slides. This hierarchical approach ensures precise diagnosis across varying spatial resolutions, addressing critical challenges in clinical assessment. To evaluate the performance of our proposed frameworks, we utilize various metrics, such as recall, precision, F1-score, accuracy, area under the curve (AUC), and average precision (AP). Additionally, we employ techniques such as the confusion matrix and classification report. Furthermore, to analyze the results and identify the strengths and weaknesses of our models, we show an intuitive uncertainty analysis and visualizations from Grad-CAM and an attention score map.

By leveraging AI and deep learning in cytopathological slides, our study addresses the critical limitations of traditional diagnostic methods. First, it helps reduce the subjectivity inherent in the Bethesda System by excluding ambiguous categories from the classification, thereby offering more consistent and accurate diagnoses. Additionally, the frameworks also allow for the analysis of specific regions within digital pathology slides, providing detailed localized diagnostic information. Furthermore, by integrating multiple instance learning (AD-MIL), the approach aggregates information from smaller patches into more comprehensive predictions, offering scalable solutions that can handle varying region sizes within slides. Finally, the integration of explainability tools like Grad-CAM and uncertainty measurements further ensures reliability and high availability in model predictions, making these frameworks useful in other datasets.

In summary, the main challenge is overcoming the subjectivity in TBS categorization while enhancing localized diagnostic information. Therefore, our study aims to address these limitations through two deep learning frameworks: (1) a CNN-based patch-level classifier that extracts cytopathological local features and accurately predicts each region, and (2) an AD-MIL framework that aggregates patch-level predictions into robust bag-level diagnostic predictions. The following sections detail the dataset and methodologies used to achieve these objectives.

This paper is organized as follows: In Section 2, we provide a comprehensive overview of related works in the domain of thyroid cancer diagnosis using deep learning, highlighting key advancements and methodologies. Section 3 expounds upon the dataset utilized for our study, outlining the process of patch generation and image preprocessing. Subsequently, Section 4 provides a thorough exposition of both the CNN-based approach and the application of the AD-MIL framework to our task. The experimental framework is outlined in Section 5, where we detail the selection of loss functions, experimental settings, and metrics chosen for evaluating the performance of the proposed models. Following that, Section 6 shows the empirical results, presenting an exhaustive assessment of the performance of models through cross-validation accompanied by the visualization of confusion matrices, ROC curves, and PR curves. In addition, it introduces the simple uncertainty analysis of the prediction outputs generated by our proposed models and visualizes important areas in images using Grad-CAM and attention scores. In Section 7, we engage in discussion, interpreting the implications of our findings, addressing potential limitations, and highlighting avenues for future research. Section 8 encapsulates our study with a concise conclusion, summarizing the contributions of our work in the context of image classification on thyroid cytopathology.

## 2. Related Works

In the realm of thyroid cancer and lesions analysis, conventional methods using numerical features and parameters have been extensively employed in previous research. Frasoldati et al. [4] proposed the diagnostic role of computer-assisted image analysis in the presurgical assessment of thyroid follicular neoplasms. It involved the analysis of cellular features, such as the ploidy histogram, proliferation index, nuclear area coefficient of variation, and anisocariosis ratio, to distinguish between benign and malignant nodules. Similarly, Gupta et al. [5] aimed to distinguish papillary and follicular neoplasms of the thyroid. They analyzed 60 cases using quantitative subvisual nuclear parameters with an image analysis system. Murata et al. [6] analyzed chromatin texture to detect malignancy in aspiration biopsy cytology, while Karslıoğlu et al. [7] used statistical approaches to examine geometric nuclear features, providing insights into how subsets of extreme values can simulate the morphological examination process. Furthermore, Aiad et al. [8] conducted an objective morphological analysis to differentiate between benign and malignant thyroid lesions using nuclear morphometric parameters. The results showed that quantitative measurements of nuclear parameters could accurately predict neoplastic nature in thyroid lesions. Other works, such as those by Tsantis et al. [9] and Ozolek et al. [10], introduced advanced classification schemes. The authors of [9] used morphological and wavelet-based features to evaluate the malignancy risk of thyroid nodules in ultrasound images. Furthermore, [10] utilized an optimal transport-based linear embedding method and existing classification methods to distinguish between follicular lesions of the thyroid based on nuclear morphology. However, these methods rely on manual feature engineering and subjective interpretation, which can be time-consuming and prone to error.

Thyroid image analysis using traditional methods gradually evolved through attempts to diagnose and classify cancer through machine learning-based image recognition. Daskalakisa et al. [11] developed a multi-classifier for distinguishing between benign and malignant thyroid nodules using the k-nearest neighbor (KNN), the probabilistic neural network (PNN), and Bayesian classifiers. The proposed system achieved a high classification accuracy of 95.7%. Wang et al. [12] presented a method for detecting and classifying thyroid lesions, specifically follicular adenoma, follicular carcinoma, and normal thyroid, based on nuclear chromatin distribution in histological images. They utilized numerical features, a support vector machine (SVM), and a voting strategy to classify the nuclei sets. Gopinath et al. [13] developed a diagnostic system for thyroid cancer diagnosis using FNAC images. The system used statistical texture features and an SVM classifier and achieved a diagnostic accuracy of 96.7%. Margari et al. [14] explored the use of classification and regression trees (CARTs) for evaluating thyroid lesions. They constructed two CART models, one for predicting cytological diagnoses and the other for predicting histological diagnoses, based on contextual and cellular morphology features. Maleki et al. [15] used the SVM to distinguish the classic PTC from noninvasive follicular thyroid neoplasm with papillary-like nuclear features and the encapsulated follicular variant of the papillary thyroid carcinoma. The SVM successfully differentiated the classic PTC with 76.05% accuracy.

With the advancement of machine learning algorithms and computational power, deep learning has emerged as a powerful technique for automatic feature extraction and classification of pathological images. Deep learning can learn complex and high-level features from images without requiring manual feature engineering. The convolutional neural network (CNN) is a type of deep learning model that is specifically designed for image analysis [16,17]. Several CNN-based models, such as VGG-16 [18], ResNet [19], and Inception [20], have garnered significant attention due to their strong performance in image classification and feature extraction tasks. These models have revolutionized computer vision, laying the foundation for numerous studies, including the application of CNNs to histopathological and cytopathological images, where they effectively capture distinct cellular characteristics and pathologies [21,22,23,24,25]. Kim et al. [21] proposed a deep semantic mobile application designed for cytopathology, specifically for the diagnosis of thyroid lesions and diseases. The application utilized Random Forest (RF), linear support vector machines (SVMs), K-nearest neighbor (KNN), and CNN for feature extraction and classification, and it outperformed hand-engineered features. Sanyal et al. [22] proposed the use of CNNs to differentiate PTC from non-PTC nodules using 512×512 pixel images from cytology smears. They demonstrated the accuracy of CNN-based thyroid cytopathological image analysis and potential applications in clinical practice. Moreover, Guan et al. [23] utilized CNNs to differentiate between PTC and benign thyroid nodules using cytological images and achieved high accuracy rates of 97.66% and 92.75% for VGG-16 and Inception-v3, respectively. Wang et al. [24] investigated the potential of CNNs to improve diagnostic efficiency and interobserver agreement in classifying thyroid nodules based on histopathological slides. The VGG-19 model demonstrated successful classification of various thyroid carcinomas, achieving excellent diagnostic capability for malignant types with accuracy of 97.34%. Additionally, Elliott Range et al. [25] developed a CNN-based machine learning algorithm (MLA) to analyze whole-slide images of thyroid fine-needle aspiration biopsies (FNABs). The performance of the MLA in predicting thyroid malignancy was comparable to that of expert cytopathologists.

Analysis of thyroid cytopathological slides poses several challenges, such as high variability and complexity due to the size of the whole-slide images as well as the lack of sufficient and reliable labels. Patch-based methods have been employed to address these issues, where large, high-resolution images are divided into smaller patches for efficient processing. This approach not only reduces computational overhead but also allows for a focus on local features, resulting in improved performance in tasks such as classification and detection [24,25,26,27,28]. However, a major limitation of patch-based methods is the lack of patch-level labels. Since patch labels are often inferred from image-level annotations, this can introduce noise and ambiguity, reducing the overall reliability and precision of the analysis. Moreover, generating precise labels for every is highly labor-intensive and time-consuming, making it impractical for large-scale datasets. To overcome these limitations, prominent directions have emerged in recent years, namely, weakly supervised learning (WSL) and multiple instance learning (MIL). WSL reduces the annotation effort by relying on less detailed patch-level labeling, such as bag-level or image-level labels, and leverages unlabeled data to enhance the model’s robustness and generalization capabilities. MIL is a type of the WSL that deals with data that are organized into bags of instances, where only the bag-level labels are available. This approach is particularly effective for capturing global context and integrating localized features into higher-order representations. This can handle the uncertainty and ambiguity of the labels and identify the relevant patterns within the bags. In particular, attention-based MIL extends this concept by incorporating an attention mechanism, which learns the importance of each instance in a bag and aggregates them into a comprehensive bag-level feature representation [29,30,31,32,33,34].

Consequently, recent research has shown a growing interest in applying MIL to the analysis of thyroid cytopathology, particularly for distinguishing between benign and malignant lesions [35,36,37]. Dov et al. [35] focused on preoperative prediction of thyroid cancer using ultra-high-resolution whole-slide cytopathology images. They proposed a CNN architecture that distinguishes between informative cytology regions and irrelevant background, training the model with both malignancy labels and TBS categories. They aggregated local estimates into a single prediction of thyroid malignancy and simultaneously predicted thyroid malignancy and a diagnostic score assigned by a human expert. Similarly, Qiu et al. [36] proposed an MIL framework using an attention mechanism with multi-scale feature fusion based on CNNs. The researchers utilized whole-slide images and identified key areas for classification without the fine-label data. The method achieved a high accuracy of 93.2% on the thyroid cytopathological data and outperformed other existing methods. Dov et al. [37] addressed machine learning-based prediction of thyroid malignancy from cytopathological whole-slide images. They proposed a maximum likelihood estimation (MLE) framework and a two-stage deep learning-based algorithm to handle the unique bag structure in cytopathology slides with sparsely located informative instances. The algorithm identified informative instances and incorporated them into global malignancy prediction. These research trends in analysis of thyroid cytopathological images advance the frontiers of medical diagnosis and enhance the clinical utility of deep learning-based tools.

## 3. Data

### 3.1. Data Collection and Preprocessing

We collect 187 whole-slide images (WSIs) for classification with TBS categories [38]. The WSIs are obtained from Wonju Severance Christian Hospital and Seoul Severance Hospital and scanned using an Olympus VS200 Digital Slide scanner (SlideView) at 20× magnification. They are stored in the Olympus VSI format and labeled by two expert pathologists. Of the 187 slides, those labeled as TBS categories III (Atypia of Undetermined Significance) and V (Suspicious for Malignancy) are excluded from our study due to their high subjectivity and diagnostic variability. The remaining 151 slides are classified into four types from TBS category: I, Insufficient for diagnosis: II, Benign; IV, Follicular neoplasm; and VI, Malignant (Papillary carcinoma).

### 3.2. Patch Extraction and Filtering

**Figure 1:**
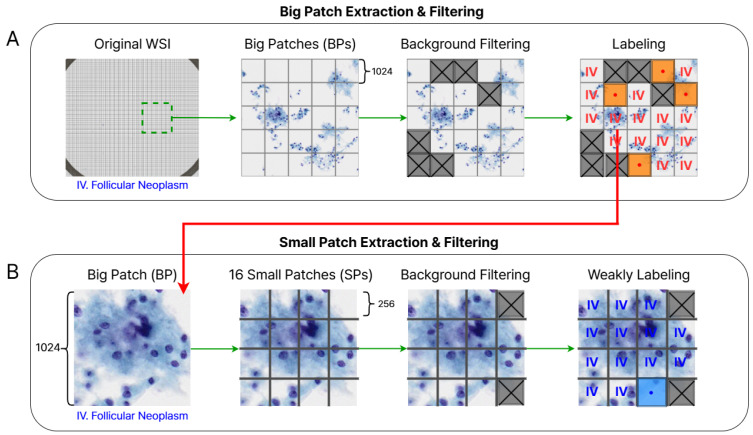
An overview of the patch extraction and filtering process for two types of datasets.

We create two patch-level datasets with different sizes, which we refer to as big patch (BP) and small patch (SP). The process of generating these datasets is described as follows (see also Figure 1):

A–1. We divide high-resolution WSIs into BP images of 1024×1024 size without overlapping.
A–2. To delete the background BPs, we convert the BP images from BGR to RGB, followed by grayscale conversion, blurring, and binary thresholding by a threshold value of 127. We discard the patches that have a ratio of black pixels lower than 1% in the binary image, as they are considered as background areas that do not contain cells.
A–3. The remaining BP images are reviewed by two cytopathologists and manually deleted if they are not consistent with the WSI-level label or belong to another category.
B–1. To reduce the computational cost during the training process, we split each BP image into 16 SP images of 256×256 size.
B–2. We apply the same process of background patch removal as described in A–2.
B–3. We also manually remove some diagnostically irrelevant patches, such as those containing only pen marks or defects without any cellular structures. The remaining SPs are used for the deep learning experiment without any additional labeling.

**Figure 2:**
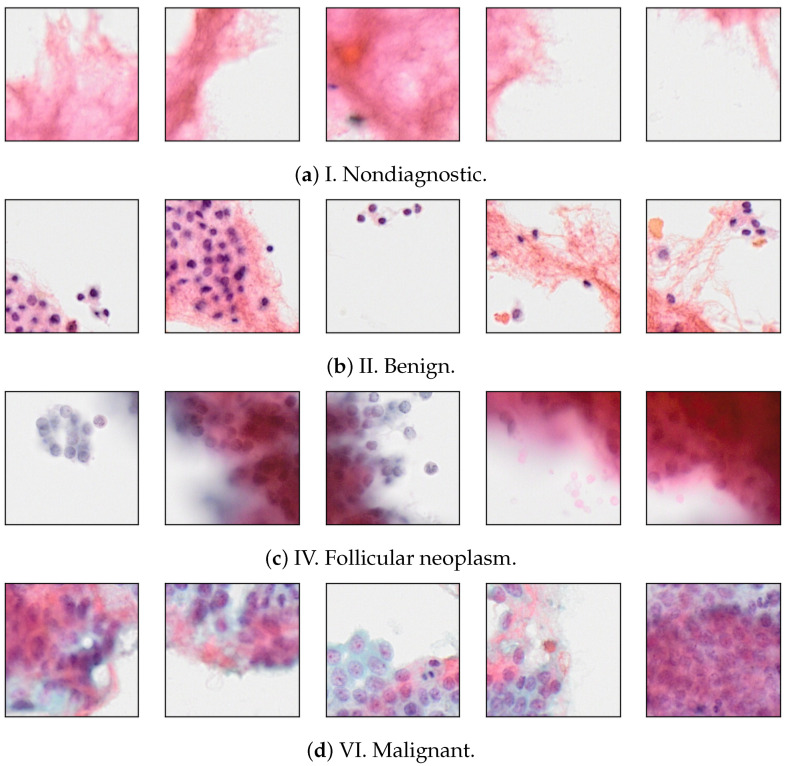
Visualization of small-patch (SP) images for each category.

The total number of BPs is 32,149, and the number of SPs is 274,594. The label distributions for each category are shown in Table 1 and Table 2, respectively. Figure 2 shows some samples of SPs for four Bethesda categories.

### 3.3. Dataset Partitioning and Normalization

We separate our dataset into a train and a validation set with a ratio of 8:2 by stratified sampling. The method improves model generalization across all classes and enables the capture of patterns even within infrequently occurring classes. The distribution of each class is preserved during dataset partitioning, ensuring similar class ratios within all sets. In the image preprocessing step, we simply convert the patch images into JPEG format and then normalize them to values between 0 and 1.

## 4. Methodologies

This paper presents patch-level classifications of two types of datasets by a convolutional neural network (CNN) architecture and an attention-based deep multiple instance learning (AD-MIL) architecture. Specifically, our custom CNN architecture, referred to as “Thyroid Cytopathology-specific CNN (TCS-CNN)”, excels at classifying small-patch images divided from cytological WSIs into 4 Bethesda categories. The TCS-CNN model is tailored for accurately identifying small regions of thyroid lesions and effectively discerning malignancy-related information, offering significant advantages in patch-level classification tasks.

### 4.1. SP Classifier Using TCS-CNN Architecture

Consider the lack of accurate labels for SP images (256 × 256 size) due to highly labor-intensive labeling, we assume them as having the same labels as the BP images (1024×1024 size). The SP dataset, including the weakly annotated SP-level label, is denoted as

$$\begin{array}{c} {\left\{X_{i},{\tilde{y}}_{i}\right\}}_{i=1}^{N_{I}}, \end{array}$$

where a small patch $X_{i}\in R^{256\times 256\times 3}$ and a weakly annotated label $\tilde{y_{i}}\in \left\{0,1,2,3\right\}$ for multi-class classification of PTC based on TBS categories. $N_{I}$ refers the number of SP-level data (=274,594).

The CNN is a type of neural network that possesses a specialized structure capable of extracting visual features from image data. It is characterized by its ability to perform convolutions, enabling it to capture local patterns and spatial relationships in the input image. To classify thyroid cytopathology images, employing CNNs presents notable advantages [21,22,23,24,25]. We build a CNN-based classifier to distinguish the PTC into four categories.

Typically, a CNN is composed of elements such as convolutional layers, pooling layers, and fully connected layers. Convolutional layers apply filters (kernels) to an input image with a convolution operation, allowing them to extract local features and spatial patterns as a feature map. Pooling layers downsample the spatial dimensions of feature maps, effectively serving to mitigate overfitting, emphasizing visual representations. Finally, fully connected layers establish interconnections between neurons, enabling complex feature learning and multi-class classification.

Among the various convolution operation-based approaches explored, a lot of advanced architectures, such as residual blocks, inception blocks, and squeeze-and-excitation blocks, are implemented and evaluated through ablation studies to gain insights necessary for building networks specialized in accurately recognizing malignant thyroid cell structures. Incorporating those CNN variants into the Conv–Pool architecture does not result in any notable performance improvements, suggesting that such modifications may not be well suited for this dataset or tasks. In contrast, simpler architectures, with standard convolution and pooling layers, achieve better performance. Specifically, configurations with 64 to 512 nodes per layer and 4 to 6 convolutional layers yield a stable performance on our SP dataset. In addition, we use both pooling techniques, max pooling and global average pooling (GAP). The max-pooling layer following each convolutional layer extracts the maximum value within each pooling region. In a similar way, the GAP layer computes the average of all elements in each feature map.

Finally, our TCS-CNN model consists of 5 convolutional layers, 5 max-pooling layers, a global average pooling (GAP) layer, and 3 fully connected layers. The filter size in all convolutional layers is 3×3. All the convolutional blocks contain a convolutional layer followed by ReLU activation function and a max-pooling layer. The two fully connected layers are used with the ReLU activation function. The last layer is a softmax layer for the multi-class classification task and it is given by

(1) $$\begin{array}{c} \mathrm{Softmax}\left(x_{i}\right)=\frac{e^{x_{i}}}{\sum_{j}e^{x_{j}}}. \end{array}$$

To prevent overfitting, He initialization [39] is applied to initiate all the trainable parameters in convolutional layers and fully connected layers, and L2 regularization and Dropout regularization are utilized for the fully connected layers.

**Figure 3:**
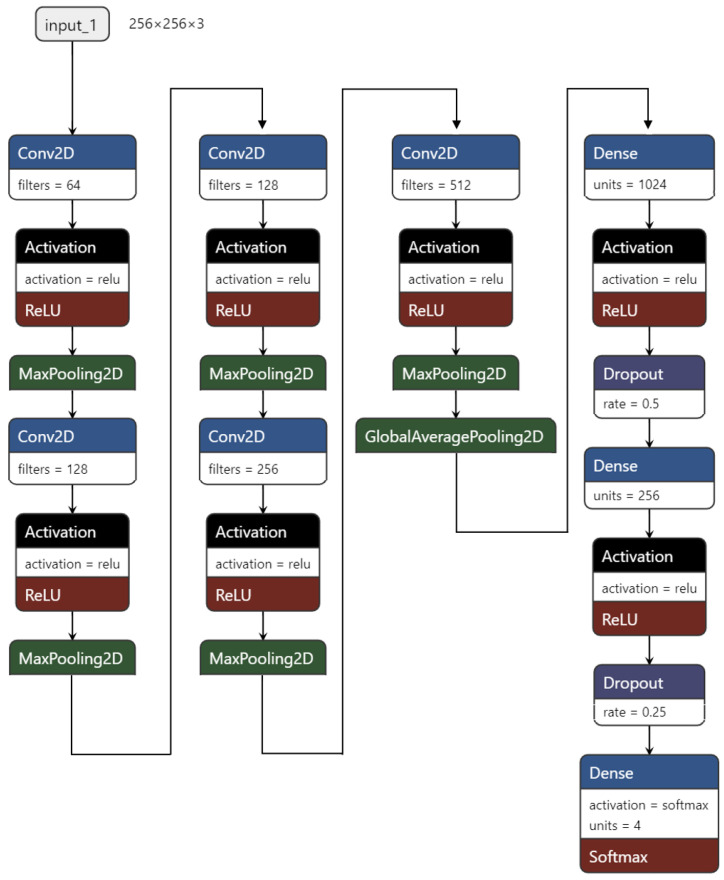
TCS-CNN architecture.

Within the TCS-CNN architecture, increasing/decreasing the number of layers or nodes generally leads to a decline in performance, indicating that the TCS-CNN structure is a well-balanced and simple design for this specific task. The detail architecture design of our proposed TCS-CNN is shown in Figure 3 below. In all Conv2D layers, the same padding, a kernel size of (3,3), and a stride of (1,1) are employed. Additionally, all MaxPooling2D layers use the same padding, with a pool size and stride of (2,2).

### 4.2. BP Classifier Using AD-MIL

The weakly labeled data used in Section 4.1 may lead to potential inaccuracy in the classification performance, either underestimating or overestimating it, thereby introducing biases into the model’s predictions. This issue arises because the SP images were generated by retiling each big patch into 16 smaller regions, and the category assigned to each SP was inherited from the corresponding BP image (i.e., weakly annotated label). For instance, SP images that do not fully encompass the lesion area within the BP still retain the same label of the BP. This discrepancy may introduce noise into the training data, potentially affecting the model’s ability to train precise and localized patterns. Hence, we overcome this problem by adopting multiple instance learning (MIL) to take the bag-level analysis using instance-level small patches.

In MIL, the dataset consists of bags and instances. The concept of a bag-level dataset in MIL involves aggregating instances into groups called bags, where the label is assigned to the entire bag rather than individual instances being assigned labels.

The MIL aggregates each instance-level prediction into a bag-level decision. Firstly, instance-level embeddings are generated through a deep learning network, capturing meaningful features. Subsequently, a pooling operation combines these embeddings into a comprehensive bag-level representation, which encapsulates the essential elements latent in instances. In this process, we utilize attention-based deep multiple instance learning (AD-MIL) to which the attention mechanism [32,40,41] is applied in the pooling operation. In AD-MIL, the bag-level feature is calculated from the weighted average of instance embeddings, where the weights reflect the importance of each instance obtained by the attention mechanism. It enables the model to de-emphasize less important ones, thereby effectively capturing the relationships between instances. The bag-level representation is fed into a classifier, such as a neural network. The network is trained by iteratively adjusting the parameters, which are shared across multiple inputs, to minimize prediction errors using bag-level labels.

**Figure 4:**
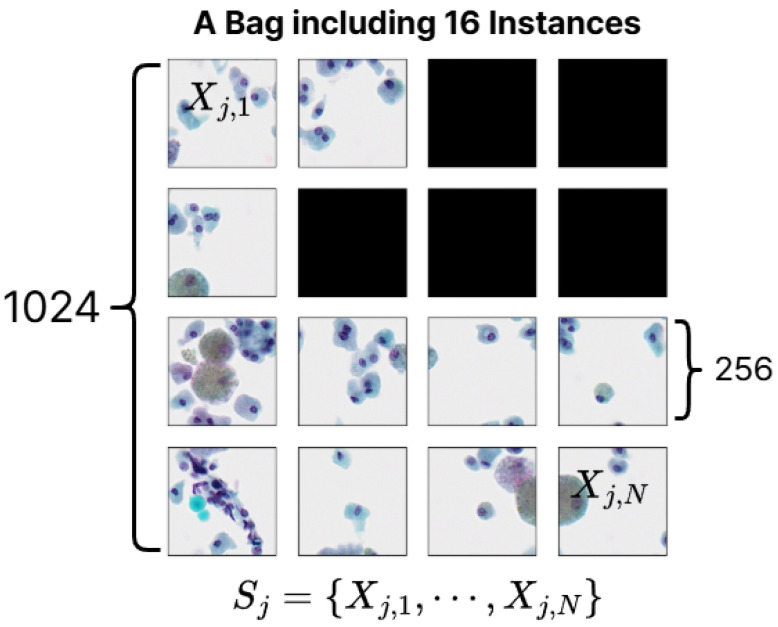
Instance-level small-patch (SP) images and padded 0-value matrices in a bag. Each bag contains 16 instances.

We take a label for each BP image as a bag-level label, and each bag contains 16 instances (SP images). Therefore, the bag-level dataset can be represented as follows:

(2) $$\begin{array}{c} {\left\{S_{j},Y_{j}\right\}}_{j=1}^{N_{B}} \end{array}$$

where $S_{j}=\left\{X_{j,1},\cdots ,X_{j,N}\right\}$ for $X_{j,n}\in R^{256\times 256\times 3}\;\;\left(n=1,\cdots ,N\right)$. A bag-level label $Y_{j}$ is in {0,1,2,3}, and $N_{B}$ is the number of bag-level data (=32,149). *N* is the number of instances in a bag, and we set N to 16 (since a 1024×1024 sized BP image is tiled into 16 smaller 256×256 images). However, as mentioned in Section 3, since some instance-level patches have been removed, the empty instance position in a bag is padded with a 0-value matrix. The process of constructing a bag-level dataset in MIL, as described, is illustrated in Figure 4.

We utilize (non-pretrained) TCS-CNN architecture in Section 4.1 as a feature extractor on MIL framework to extract low-dimensional representations from the instance-level patch images. Furthermore, the attention networks combine these instance-level representations $h_{n}\left(n=1,\cdots ,N\right)$ into bag-level representations z, weighing the key instance SPs that have a major impact on thyroid malignancy prediction. This MIL is formulated as Equation (3). The attention scores $a_{n}\left(n=1,\cdots ,N\right)$ show the importance of each instance-level patch, and we use the gated attention mechanism [32] denoted as Equation (4) below:

(3) $$\begin{array}{c} z=\sum_{n=1}^{N}a_{n}h_{n}, \end{array}$$

(4) $$\begin{array}{c} a_{n}=\frac{\exp\{w^{T}\left(\tanh\left(Vh_{n}^{T}\right)⊙\sigma \left(Uh_{n}^{T}\right)\right)\}}{\sum_{j=1}^{N}\exp\left\{w^{T}\left(\tanh\left(Vh_{j}^{T}\right)⊙\sigma \left(Uh_{j}^{T}\right)\right)\right\}}, \end{array}$$

where $w\in R^{L\times 1}$, $V\in R^{L\times D}$, and $U\in R^{L\times D}$. *L* is a dimension of weights and *D* is a dimension of the instance-level feature vector. Additionally, ⊙ represents a Hadamard operator for the element-wise multiplication and σ is the sigmoid function. The utilization of a sigmoid gate in the gated attention mechanism enables the capture of interactions among instances with varying strengths, facilitating the extraction of more sophisticated information.

**Figure 5:**
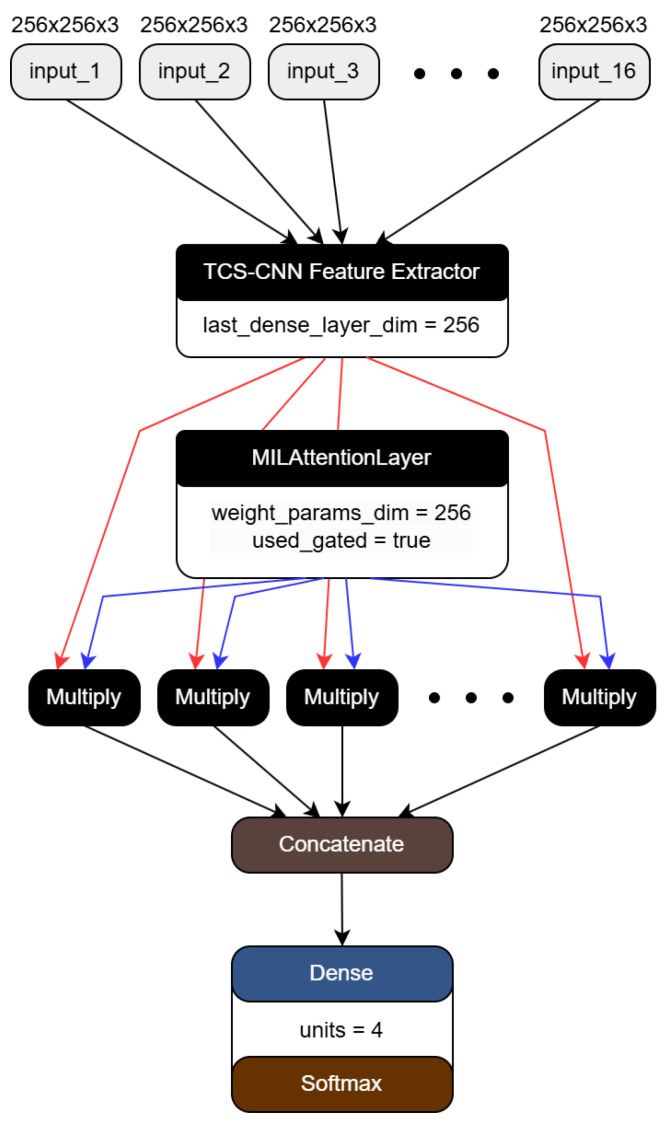
The AD-MIL architecture. The red arrows represent the features generated by the TCS-CNN, while the blue arrows indicate the attention weights.

These bag-level embeddings are concatenated, and they are classified by a fully connected layer with the softmax activation. Figure 5 shows the architecture of the MIL, which has 16 inputs.

## 5. Experiment

Our two classification models are trained and evaluated on the two datasets mentioned in Section 3. The inputs of TCS-CNN and AD-MIL are the instance-level (SP-level) dataset and bag-level dataset, respectively. These proposed methods are evaluated in comparison with pretrained CNN models with ImageNet [42], which are VGG16 [18], Inception-v3 [20], and Mobilenet [43].

For each of these pretrained models, we perform an ablation study to identify the best configuration by freezing all layers and progressively unfreezing them from the final layer to the initial layer, allowing each layer to become trainable. The performance is evaluated at each stage, and the models with the highest validation recall scores are selected. We apply the training process and evaluation settings in the same way as the TCS-CNN experiment in Section 4.1 to these comparative models.

### 5.1. Training Procedure

Due to the unbalanced distribution of the classes, we use the weighted cross-entropy (WCE) loss function. As shown in Table 2, the class imbalance is indicated by the class weight ratio, which ranges from 0.48 to 2.95 for the small patches, indicating that the least represented class has approximately six times fewer samples than the most dominant one. During the computation of the loss function, the WCE can confer higher weights upon the minority classes while endowing the majority classes with comparatively lower weights. The weights are inversely proportional to the size of the class containing the *i*-th sample, as shown in Equation (5) below.

(5) $$\begin{array}{cc} \mathrm{WCE}(y_{ij},{\hat{y}}_{ij}) & =-\sum_{i=1}^{M}\sum_{j=1}^{C}w_{j}y_{ij}\log\left({\hat{y}}_{ij}\right), \end{array}$$

$$\begin{array}{cc} w_{j} & =\frac{M}{N_{j}\times C}, \end{array}$$

where *M* is the number of samples and *C* is the number of classes. Additionally, $N_{j}$ represents the number of *j*-th class samples. $w_{j}$ corresponds to the weight ascribed to class *j*, and this weight is computed to be inversely proportional to the frequency of the class.

For training the networks, the Adam optimizer [44] is chosen with an adaptive learning rate that decreases when a validation recall stops increasing. The learning rate starts with 0.0001 and decays by a factor 0.8 with 3-patience. It ensures stable convergence by mitigating overfitting through controlled learning rate adjustments. Furthermore, the automated regulation of the learning rate during training streamlines the hyperparameter tuning, contributing to improved model generalization by prioritizing stability.

The epoch is set to 50 and the batch size is set to 8. We select the model with the best recall score for the validation set during training. All experiments are conducted in the same environment using the same hyperparameters and optimization method and implemented in Python 3.8.19 with Tensorflow 2.9.0. All models are trained on an NVIDIA GeForce RTX 4080 GPU with 16 GB of memory.

### 5.2. Evaluation Metrics

We utilize the accuracy, precision, recall, and f1-score metrics to compare the performance of the models. The metrics are represented as follows:

$$\begin{array}{cc} \mathrm{Precision} & =\frac{TP}{TP+FP},\\ \mathrm{Recall} & =\frac{TP}{TP+FN},\\ \mathrm{Accuracy} & =\frac{TP+TN}{TP+TN+FP+FN},\\ f1-\mathrm{score} & =\frac{2\times \mathrm{Precision}\times \mathrm{Recall}}{\mathrm{Precision}+\mathrm{Recall}}, \end{array}$$

where TP represents the true positive, FP is the false positive, and TN and FN are the true negative and false negative, respectively. Accuracy quantifies the overall correctness of predictions of the model, but it can be misleading for imbalanced datasets. Therefore, we analyze the following three metrics together. Precision focuses on the accuracy of positive predictions, and recall assesses how well the model accurately makes predictions belonging to the positive class. A high precision ensures reliable identification of positive cases, minimizing unnecessary treatments, while a high recall guarantees thorough recognition of cancer. In addition, f1-score offers a balanced evaluation of the performance of the model by harmonizing precision and recall.

**Figure 6:**
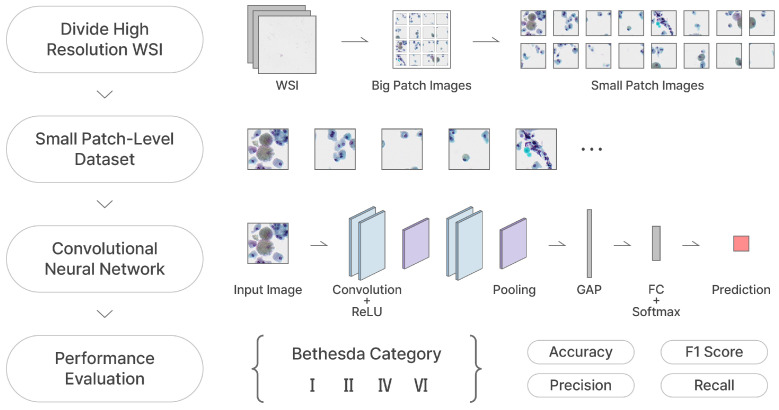
TCS-CNN training process.

**Figure 7:**
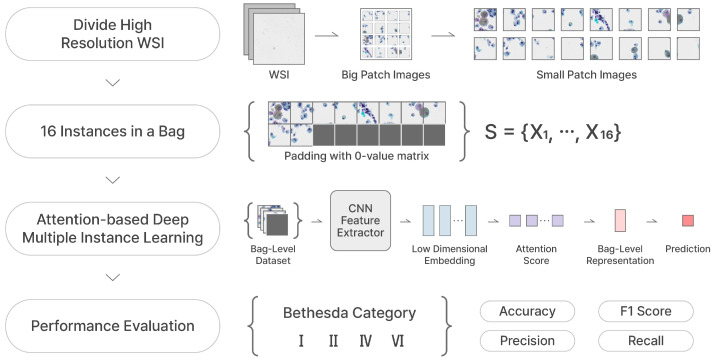
AD-MIL training process.

Our two proposed frameworks are represented in Figure 6 and Figure 7, respectively. We crop the WSIs into small patches of 256×256 size and feed them into the TCS-CNN model for four-class classification. Prior to global average pooling (GAP), meaningful feature maps are obtained from convolutional blocks including convolution, ReLU, and pooling. After that, the fully connected (FC) layers are used to integrate global information and learn parameters for the final prediction. In the AD-MIL framework, as shown in Figure 7, we generate a bag containing 16 instances and utilize it as the input of the AD-MIL training process. The TCS-CNN feature extractor trains the features for instances, and their significance in a bag is applied to the classification by the attention score. Through the two processes, two types of dataset are classified into four Bethesda categories, and finally, we use four metrics to conduct the quantitative evaluation.

## 6. Results

### 6.1. Performance

**Figure 8:**
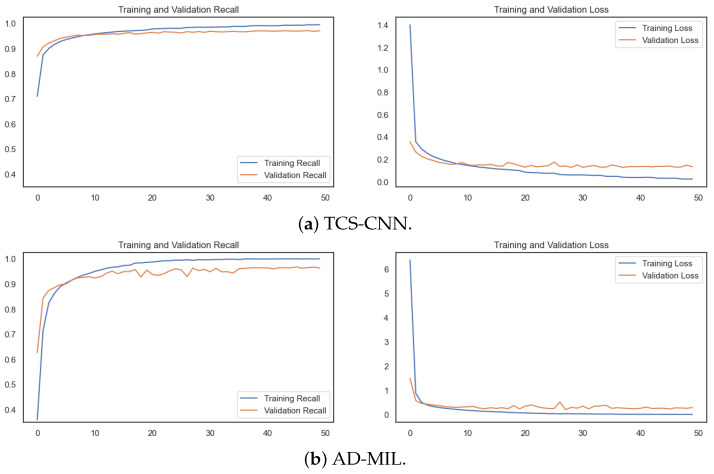
The training history of recall and loss on the train/validation sets.

As shown in Figure 8, the training convergence is achieved before 50 epochs. Both the training and validation loss consistently decrease, indicating the model rapidly adapted to the intricacies of the AI task. Importantly, the recall metrics for both sets show a consistent increase, further supporting the model’s capability.

Model performance is evaluated on the validation set using accuracy, precision, recall, and f1-score. In addition, the receiver operating characteristic (ROC) curve and precision–recall (PR) curve are utilized for comprehensive and accurate assessment of performance. Table 3 represents the quantitative evaluation of the TCS-CNN by using stratified k-fold cross validation. This validation method is a technique where the dataset is split into *k* equally sized folds, ensuring that each fold has the same proportion of samples from each class as the original dataset. It is performed with the ratio of 8:2, as mentioned in Section 3, and this allows us to make better use of all of the dataset while providing confidence in model outcomes. Overall, our proposed TCS-CNN achieves 95–96% precision, 95–96% recall, 95–96% f1-score, and 97% accuracy. All later results for the TCS-CNN and AD-MIL are for a one-split subset of the cross-validation sets.

We compare our proposed models with VGG16, Inception-v3, and Mobilenet in Table 4. The three comparison models are subjected to the same experimental setup as TCS-CNN and are also SP classifiers. The TCS-CNN, a SP classifier, and AD-MIL, which utilizes the TCS-CNN architecture as a feature extractor to perform bag-level classification, show comparable performance levels. This comparison highlights the significance of an efficient architecture. Notably, the simpler architectures of both TCS-CNN and AD-MIL outperform the other models across four metrics. The success of these models, achieved while maintaining simplicity, challenges the common belief that increasing model complexity always leads to better results.

The TCS-CNN effectively extracts meaningful features related to thyroid malignancy from SP images, enabling accurate diagnosis in small regions in large-scale slides. These extracted features, when aggregated, contribute to more accurate predictions at the bag level, demonstrating that the model enables the successful extension of diagnosis to larger-patch-level areas through the thyroid carcinoma-specific structure.

Table 5 shows the comparative classification performance of TCS-CNN and AD-MIL models for four categories. Two models exhibit commendable performance across all classes and demonstrate consistent results in terms of precision, recall, and F1-score. For the TCS-CNN model, overall performances are highest for categories I and IV, indicating the model’s proficiency in accurately identifying these classes.

Notably, the classification performed using the AD-MIL surprisingly shows improved performance in category VI. This observation underscores the success of expanding the diagnostic region and offers valuable insights into the capabilities of the model in addressing the complexities associated with the malignant case. This is achieved through its attention mechanism, which dynamically allocates importance to specific areas, thus capturing subtle features essential for accurate predictions within the malignant class.

**Figure 9:**
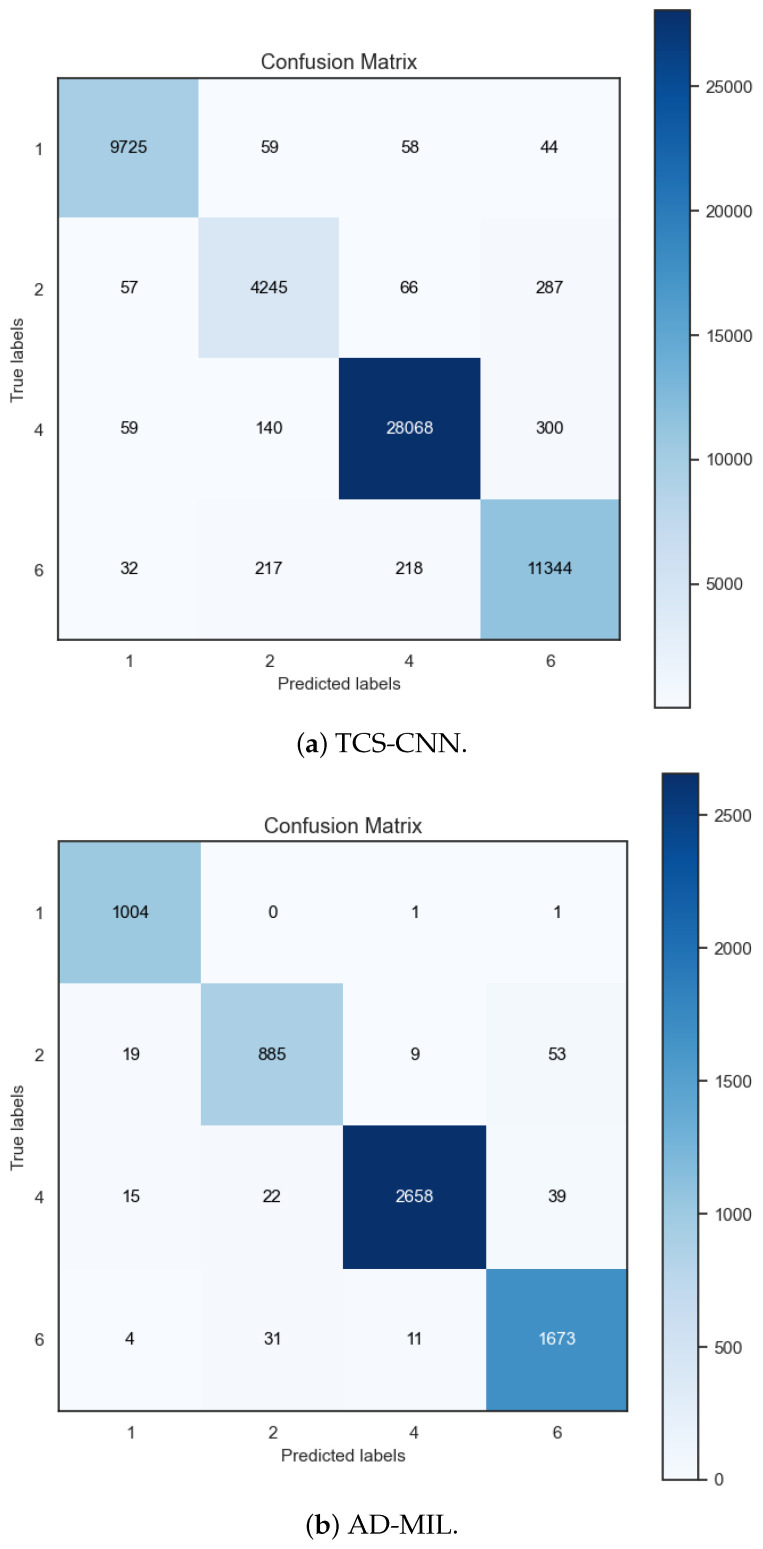
Confusion matrices of TCS-CNN and AD-MIL.

Additionally, Figure 9 shows confusion matrices of two proposed algorithms, each trained on different datasets: the TCS-CNN model trained on the SP dataset and the AD-MIL model trained on the bag-level (BP-level) dataset. The confusion matrix summarizes models’ predictions against the ground-truth labels for each class. We can see that each confusion matrix exhibits high values predominantly along its main diagonal. This pattern signifies that both models possess a remarkable proficiency in correctly predicting outputs within their respective classes.

**Figure 10:**
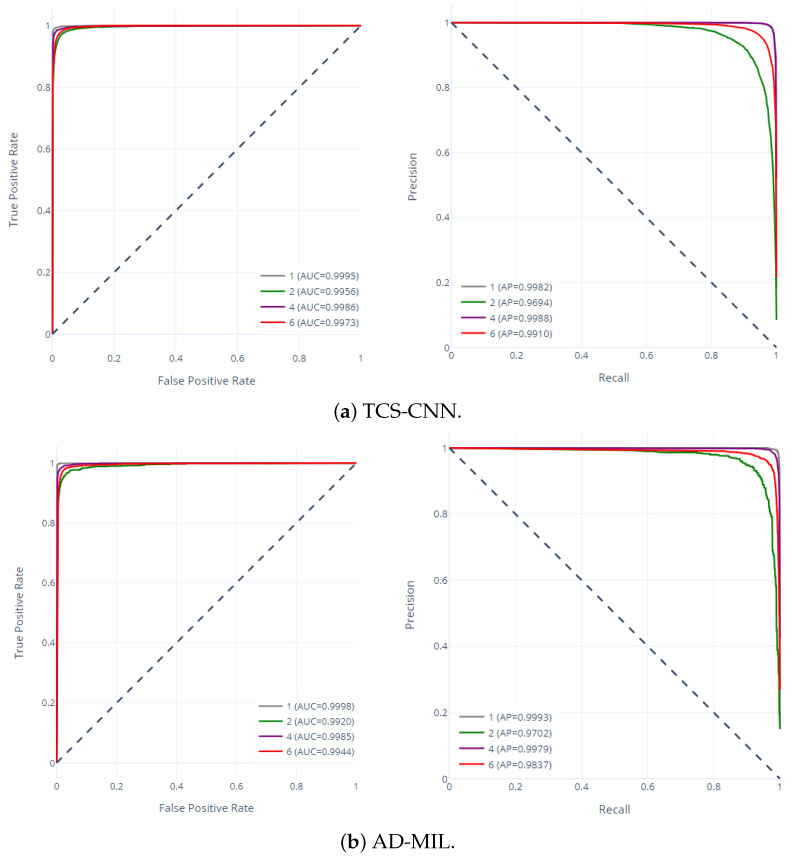
ROC curves and PR curves of TCS-CNN and AD-MIL.

The receiver operating characteristic (ROC) curve and precision–recall (PR) curve for two models are shown in Figure 10. The ROC curve shows a representation of the trade-off between the true-positive rate and the false-positive rate and provides understanding of a model’s ability to discriminate between classes. Moreover, the PR curve represents the relationship between precision and recall, offering perspective on a model’s performance in capturing true positives while minimizing false positives. The high area under the curve (AUC) values, ranging from 0.99 to 1.0, emphasize the impressive discriminatory capabilities of both models across all classes. Similarly, the elevated average precision (AP) values affirm the models’ efficacy in accurately identifying true-positive instances while maintaining low false-positive rates. These exceptional and highly accurate performances highlight their potential for real-world applications in medical diagnostics and research.

In addition, we analyze confidence distributions from TCS-CNN predictions to understand uncertainty and to obtain reliability of the model. We observe that over 97% of the confidence values generated by the TCS-CNN for all small patches are greater than 0.9. This suggests that the model confidently classifies each patch into one of the four Bethesda categories. However, this can also reflect the overconfident nature of modern neural networks [45,46]. Therefore, it is essential to check the uncertainty of these patch-level predictions to ensure that the model is indeed making reliable diagnoses.

### 6.2. Uncertainty Analysis

To better understand the predictions from TCS-CNN and evaluate the reliability of its output, we can calculate the uncertainty inherent in each patch-level prediction. Neural networks, especially deep models like CNNs, can generate highly confident predictions for input patches. However, a high confidence value does not necessarily indicate accuracy, and it is crucial to quantify the uncertainty associated with these predictions. Uncertainty analysis plays a pivotal role in assessing how much trust can be placed in the model’s outputs, particularly when dealing with rare or ambiguous cases. In this section, we focus on measuring the uncertainty of the TCS-CNN’s predictions and offer a deeper understanding of how confident the model is about its decisions [45,46].

To address the high confidence values produced by TCS-CNN, we aim to directly and intuitively assess the uncertainty associated with each patch’s prediction. As described in Section 4.1 and illustrated in Figure 3, the final layer of TCS-CNN is a fully connected layer with 256 nodes. A single output of this layer, denoted as $Z\in R^{256}$, feeds into the softmax function Equation (1) to compute category predictions, represented as Softmax(Z)∈{0,1,2,3}.

By leveraging the final hidden layer activations Z, we calculate $U_{\max}$ and $U_{\mathrm{entropy}}$, providing a measure of uncertainty for each patch. $U_{\max}$ is the negative value of the confidence value, and a higher $U_{\max}$ indicates lower uncertainty. Similarly, $U_{\mathrm{entropy}}$ applies the logarithm to each class probability and computes the weighted sum of these values, yielding a negative value for uncertainty. Higher uncertainty corresponds to increased entropy, making it useful for capturing various possibilities in the patches.

$$\begin{array}{c} U_{max}\left(z\right)=-\max_{i}\psi {\left(Z\right)}_{i}\;\mathrm{and}\;U_{entropy}\left(Z\right)=-\sum_{i=1}^{C}\psi {\left(Z\right)}_{i}\log\psi {\left(Z\right)}_{i}, \end{array}$$

where ψ is the softmax function and *C* is the number of classes (=4).

**Figure 11:**
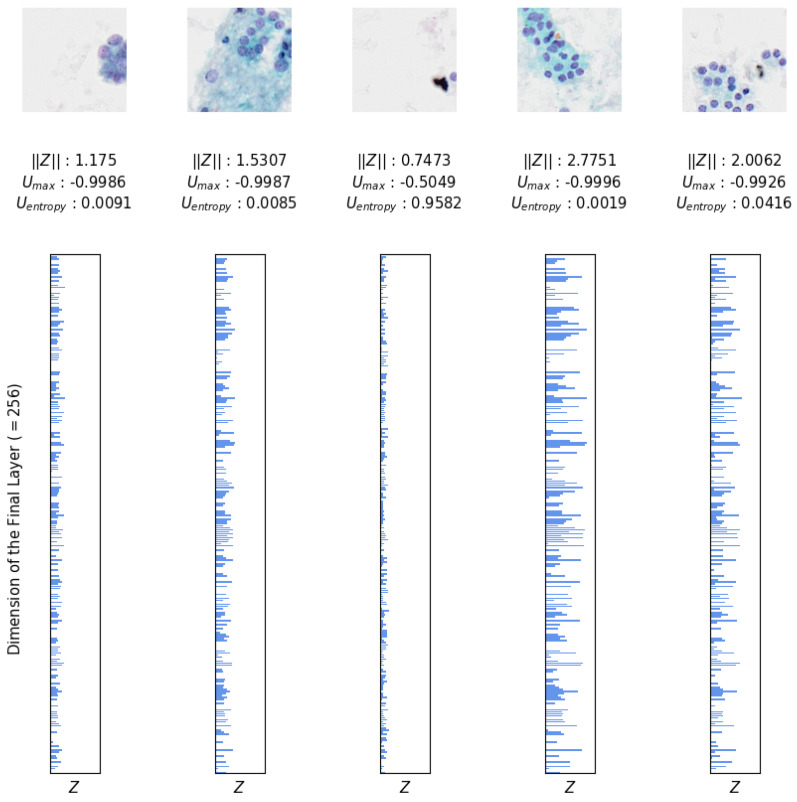
Uncertainty measurements of 5 validation data (SP images) generated by TCS-CNN, inspired by the figure presented in [46].

Additionally, the magnitude of Z(=||Z||) is used to further analyze and quantify the confidence associated with TCS-CNN’s outputs. TCS-CNN is effectively trained using only the patches that play a significant role in diagnosis within the slide. Therefore, when patches that are irrelevant to the diagnosis feed into the model, the uncertainty associated with these patches increases. This indicates that the model recognizes these patches as not contributing meaningfully to the diagnosis and therefore has lower confidence in its predictions. Therefore, by observing ||Z||, we can infer how diagnostically relevant, meaningful, and indicative of ROI (Region of Interest) a given patch image is. The larger the magnitude of the final layer’s output, the more important it is, and this greatly influences the subsequent calculation of softmax probabilities. These measurements are illustrated in Figure 11, providing insights derived directly from the outputs of the strongly trained classifier, enabling an interpretation of its confidence and uncertainty for each patch space.

### 6.3. Visualization

Gradient-weighted class activation mapping (Grad-CAM) is widely employed for visual interpretation of CNN-based deep learning models [47]. It elucidates the important regions within an input image that contribute significantly to a particular network’s classification decision. In more detail, Grad-CAM operates on the gradients of the target class’s score with respect to feature maps of the final convolutional layer and assigns relevance scores to individual spatial locations by analyzing these gradients, thereby highlighting areas that strongly influence the network’s prediction. In the context of utilizing TCS-CNN and Grad-CAM for the classification of thyroid cytopathological images into Bethesda categories, it is noteworthy that the regions highlighted by Grad-CAM within the cellular constituents of the input images are likely to hold pivotal importance in the classification process.

**Figure 12:**
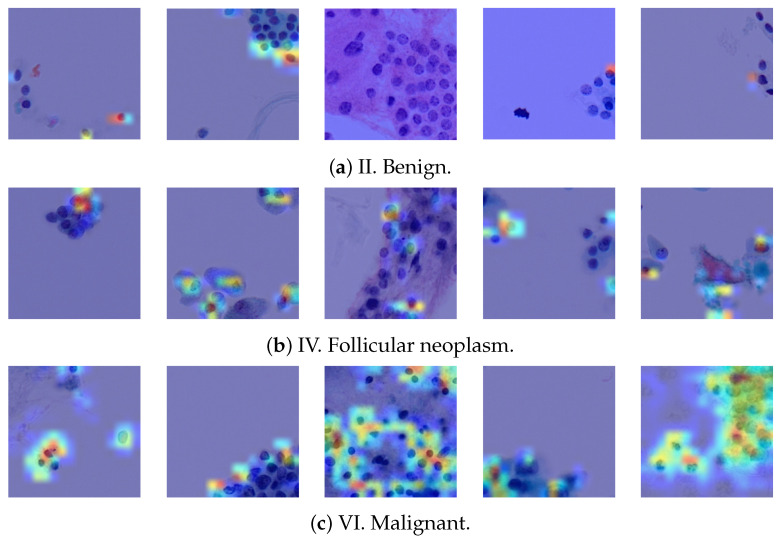
Grad-CAM heatmap visualizations.

Therefore, we show the interpretability of the proposed TCS-CNN by the Grad-CAM algorithm. Figure 12 exhibits a comprehensive visual analysis of the Grad-CAM activations for five samples from each Bethesda category II, IV, and VI. The SP images corresponding to these categories often contain cellular structures such as nuclei, which are critical for the diagnosis of thyroid cytopathology. By observing these Grad-CAM activations, it becomes evident that the network does not highlight every cellular structure indiscriminately. Instead, the activations predominantly focus on more malignant cellular features, selectively emphasizing regions indicative of higher diagnostic significance. This selective attention suggests that the TCS-CNN model effectively differentiates between benign and malignant features, showcasing a level of precision that aligns closely with the diagnostic reasoning like human pathologists.

Furthermore, we can visually examine the attention scores of AD-MIL to provide evidence that such accurate diagnoses can be extended to larger regions. The AD-MIL introduced in Section 4.2 trains the attention score values $a_{n}\left(n=1,\cdots ,N\right)$ for multiple instance patches within a bag and uses these scores to make bag-level decisions by considering the relative importance of each region.

**Figure 13:**
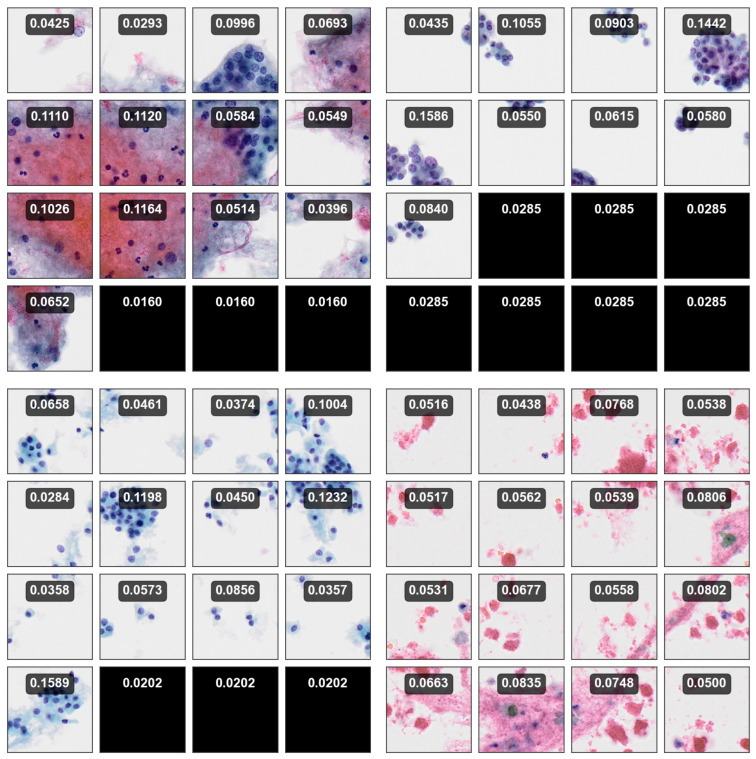
Attention score maps of AD-MIL with 4 bag-level (BP-level) images.

Figure 13 illustrates attention score maps for 4 BP images, each consisting of 16 SP images. The maps depict the importance of each SP, where higher attention scores are assigned to regions that contribute more significantly to the bag-level diagnosis. This highlights that the model can focus on regions that are more diagnostically important and relevant.

## 7. Discussion

In this paper, we present two deep learning frameworks for the classification of thyroid cytopathology images based on the TBS categories. The first framework is a CNN-based model that classifies small-patch images into four categories, and the second one is an AD-MIL model that classifies a bag of small-patch images into the same categories based on their bag-level (big-patch-level) labels. We evaluate the performance of both frameworks using various metrics, such as accuracy, precision, recall, F1-score, AUC, and AP. Furthermore, we also use confusion matrices, classification reports, simple analysis of uncertainty, and visualizations using Grad-CAM and attention scores to identify the strengths and weaknesses of the models.

Our results show that the TCS-CNN model effectively classifies small-patch images from WSIs, achieving notable performance in distinguishing between four distinct TBS categories, including benign and malignant lesions. The simplicity of the model architecture, consisting of basic convolutional layers with regularization strategies, contributes to its efficiency in classifying small patches while avoiding overfitting. The addition of AD-MIL, designed to handle weakly labeled data, represents high accuracy for larger-patch-level datasets by aggregating instance-level features into bag-level predictions using an attention mechanism. The model demonstrates the potential of such methods for large-scale medical image datasets, where manual labeling at the small-patch level is impractical, offering a more scalable and cost-effective approach to analyzing large cytopathological datasets.

Our findings are consistent with prior studies, which used CNNs and multiple instance learning for thyroid cytopathology analysis. However, we define a unit for small patches and sequentially aggregate them to create bag-level datasets, transitioning from smaller-patch units to slightly larger-patch areas. This approach demonstrates a difference from other studies that construct bag-level datasets based on whole-slide images. Furthermore, the incorporation of a simple, efficient CNN architecture is key to improving classification performance without requiring highly complex model designs.

Despite the promising results, several limitations need to be addressed. One of the primary challenges is the inherent noise introduced by weak annotations, particularly in small patches that were not fully labeled by expert cytopathologists. Although AD-MIL mitigates this to some extent, the overall quality of small-patch-level labels still impacts the model’s ability to classify instances with high precision. Moreover, while our model achieves good performance on the validation dataset, its generalizability to other datasets with different characteristics or annotation quality remains to be tested.

Despite the aforementioned limitations, our approach can be further improved by examining the uncertainty in predictions, as suggested in Section 6.2 and Section 6.3, and by monitoring attention scores to ensure the model fully understands the patches. By conducting experiments that assess these factors, we believe that addressing potential issues related to generalization when applying our approach to other cytopathology slides could be achieved. This approach may help mitigate the challenges posed by dataset variability and improve the robustness of the model across different cytopathological datasets.

Finally, future works can focus on several key areas to further improve the model’s performance and applicability. First, improving labeling techniques using semi-supervised or active learning methods can help alleviate the impact of weak annotations by dynamically refining label quality. Additionally, evaluating the generalization of models to other malignancies or cytopathological datasets, possibly through transfer learning, can enhance its versatility. Data augmentation strategies can also be explored to strengthen the robustness of models by generating synthetic samples that reflect various lesion characteristics. Similarly, enhancing model explainability, particularly identifying which patches or features contribute most to the final classification, can improve its clinical relevance and facilitate its integration into medical practice.

## 8. Conclusions

Our study presents a novel contribution to the field of thyroid cytopathology by applying artificial intelligence and deep learning techniques to analyze cytological images obtained from fine-needle aspiration cytology. We propose two deep learning frameworks, utilizing two distinct types of dataset: a small-patch-level classifier employing the TCS-CNN architecture and a bag-level (big-patch-level) classifier employing an AD-MIL framework.

Our results show that both frameworks achieve high accuracy and recall scores, demonstrating their effectiveness in distinguishing between different types of thyroid lesions. TCS-CNN achieves an accuracy of 97% and a recall of 96%, and the AD-MIL model achieves an accuracy of 97% and a recall of 96%. These results are comparable or superior to the existing methods for image analysis of thyroid cytopathology. Moreover, both frameworks outperform pretrained CNN models, such as VGG-16, Inception-v3, and Mobilenet, indicating that our custom-designed TCS-CNN model is more suitable for the task.

However, both frameworks also have some limitations and challenges that need to be addressed. The TCS-CNN relies on small-patch-level labels for small-patch images, and the weakly annotated labels are derived from the labels of big-patch images, which may not reflect the true labels of the small-patch images. The AD-MIL model overcomes this problem by using bag-level labels, which are more accurate and consistent. However, the AD-MIL model also faces some challenges, such as the handling of missing or irrelevant instances and the interpretation of attention scores. In addition, although our study did not directly perform WSI-level classification due to the limited availability of WSIs, this remains a crucial task in the field of thyroid cytopathology. We believe that our patch-level classification approach can serve as a foundation for advancing towards WSI-level diagnosis via patch aggregation methods in the future.

In summary, we have shown that both frameworks can achieve high accuracy and recall scores and can capture the relevant features and small and big regions of interest in whole-slide images. We have also discussed the limitations and challenges of both frameworks and suggested some possible directions for future research. Our work contributes to the field of thyroid cytopathology by providing novel and effective methods for the analysis of cytological images derived from FNAC, offering rapid and precise results for PTC differential diagnosis. Our work also opens avenues for enhanced patient outcomes and cost-effective healthcare solutions.

## Figures and Tables

**Table 1:** The number of big-patch (BP) images for each category.

Category	Number of Images
I. Nondiagnostic	5032
II. Benign	4832
IV. Follicular neoplasm	13,670
VI. Malignant	8615
Total	32,149

**Table 2:** The number of small-patch (SP) images for each category.

Category	Number of Images
I. Nondiagnostic	49,433
II. Benign	23,272
IV. Follicular neoplasm	142,834
VI. Malignant	59,055
Total	274,594

**Table 3:** Cross-validation results of the TCS-CNN.

Metrics	Split 1	Split 2	Split 3	Split 4	Split 5
Precision	0.9578	0.9599	0.9604	0.9622	0.9623
Recall	0.9597	0.9602	0.9568	0.9596	0.9558
F1-score	0.9587	0.9601	0.9585	0.9609	0.9589
Accuracy	0.9720	0.9727	0.9721	0.9732	0.9723

**Table 4:** Performance comparison of the proposed models and 3 pretrained models.

Model	Precision	Recall	F1-Score	Accuracy
VGG16	0.9324	0.9327	0.9326	0.9522
Inception-v3	0.8587	0.8472	0.8527	0.8842
Mobilenet	0.9259	0.9267	0.9263	0.9461
**TCS-CNN**	**0.9578**	**0.9597**	**0.9587**	**0.9720**
**AD-MIL**	**0.9616**	**0.9649**	**0.9631**	**0.9681**

**Table 5:** Classification report for 4 categories of TCS-CNN and AD-MIL.

Method	Cancer Types	Precision	Recall	F1-Score	Support
TCS-CNN	I. Nondiagnostic	0.9850	0.9837	0.9844	9886
	II. Benign	0.9107	0.9119	0.9113	4655
	IV. Follicular neoplasm	0.9880	0.9825	0.9852	28,567
	VI. Malignant	0.9473	0.9605	0.9538	11,811
	**Average**	0.9578	0.9597	0.9587	54,919
AD-MIL	I. Nondiagnostic	0.9635	0.9980	0.9805	1006
	II. Benign	0.9435	0.9161	0.9296	966
	IV. Follicular neoplasm	0.9922	0.9722	0.9821	2734
	VI. Malignant	0.9473	0.9732	0.9601	1719
	**Average**	0.9616	0.9649	0.9631	6425

## Data Availability

Some or all datasets generated during and/or analyzed during the current study are not publicly available but are available from the corresponding author on reasonable request.
